# Impact of COVID-19 pandemic on health care system, work, and mental well-being of people with cystic fibrosis

**DOI:** 10.1007/s11845-023-03391-w

**Published:** 2023-05-12

**Authors:** Rini Bhatnagar, Sarah Tecklenborg, Ricardo Segurado, Philip Watt, Naula McAuley, Patricia Fitzpatrick

**Affiliations:** 1 School of Public Health, Physiotherapy and Sports Science, University College Dublin, Dublin, Ireland; 2 Cystic Fibrosis Ireland, Dublin, Ireland

**Keywords:** COVID-19, Cystic fibrosis, Employment, Hospital visits, Mental well-being, Ireland

## Abstract

**Background:**

COVID-19 pandemic has been challenging for all, particularly for high-risk groups including people with cystic fibrosis (PWCF).

**Aim:**

This study aims to examine impact of COVID-19 pandemic on the lives of PWCF in relation to hospital visits, use of telemedicine, employment, and mental well-being.

**Methods:**

A cross-sectional online survey was developed by the Cystic Fibrosis (CF) Ireland research team and uploaded on SmartSurvey UK. The survey was advertised by CF Ireland via their website and social media in October 2020. The University College Dublin research partner team conducted the analysis. Logistic regression was used for the analysis, using IBM SPSS Version 26.

**Results:**

One hundred nineteen PWCF responded. 47.5% deferred their hospital visits, with delays ranging from 1 to 6 months. Deferrals impacted rehabilitation therapies, medical care at hospital, and diagnostic tests. For many, online consultation was a new experience (51.7%), and 87.8% were satisfied with this method. Among those who worked during lockdown (47.8%), 87.2% (*n* = 48) worked at home. PWCF aged < 35 years (9.6%) were more likely to work onsite as compared to those > 35 years (1.9%). When adjusted for gender and employment, PWCF aged < 35 years were more likely to feel “nervous” (OR: 3.28; *P* = 0.02), “nothing could cheer them up” (OR: 3.24; *P* = 0.04), and “tired” (OR: 2.76; *P* = 0.02) as compared to those > 35 years.

**Conclusion:**

COVID 19 pandemic has greatly impacted PWCF in terms of hospital visits, access to tests, CF care, and psychological well-being. Younger PWCF reported greater impact on psychological health. Online consultation and electronic prescription were welcomed and could have a role post-pandemic.

## Introduction

Cystic fibrosis (CF) is a common, inherited, autosomal recessive, life-threatening disorder that affects the respiratory and digestive systems. It is most commonly observed in Caucasians [1]. The cause is a mutation in cystic fibrosis transmembrane conductance regulator (CFTR) gene. The CFTR protein encoded by this gene functions as a chloride channel, enabling the passage of chloride ions across epithelial cell membranes. Since chloride drives water movement, CFTR plays a vital role in the regulation of water and salt balance in body fluids such as mucus and sweat. Alterations in the CFTR gene result in the production of thicker mucus throughout the body, which leads to persistent lung infections and impairs the functioning of other organs as well [2]. Recent statistics from the “European Cystic Fibrosis Society Patient Registry” show that approximately 50,000 people (both children and adults) with CF reside in 35 European nations [3]. Ireland has the highest prevalence of CF worldwide [4]. According to Cystic Fibrosis Registry Annual Report 2020, 1256 living individuals with CF reside in Ireland; 60.4% are > 18 years [5].

The World Health Organization defined SARS COV-2 as a highly infectious respiratory disease caused by a novel coronavirus [6]. The COVID-19-related public health restrictions have impacted healthcare systems worldwide. The emergence of the pandemic caused great challenges for the care of adults with CF. Although most people infected with the COVID-19 virus experienced mild to moderate respiratory disease and recovered without any special treatment, the majority of hospitalisations and intensive care admissions occurred in those who belonged to a high-risk category. Although, PWCF are more vulnerable to viral respiratory tract infections than the general population; with a novel virus, there was uncertainty about the impact on those living with CF, and data remains limited, but those on immunosuppressant treatment post-transplant were certainly deemed a particularly vulnerable group [6].

As the COVID-19 pandemic developed, most countries including Ireland introduced public health restrictions to stop viral transmission [4]. To reduce the risk, PWCF were advised to cocoon. This included a series of measures aimed at minimising all interaction between themselves and others outside their family unit since the outbreak of the COVID-19 pandemic in Ireland in late March 2020 until the start of vaccinations in 2021 [4]. Most recent guidelines advise that, once fully vaccinated, most people in a high-risk category can follow standard population public health advice. However, vaccination may not be fully effective for some people with weak immune systems or had undergone organ transplants; therefore, they are advised to continue to follow extra precautions even after being vaccinated [7].

COVID-19 caused global economic disruptions leading to furloughs and business closures, resulting in PWCF losing their jobs [8]. Delivery of CF care was greatly impacted during the pandemic. To mitigate and manage the virus, many countries worldwide implemented measures such as the closure of medical units and the redeployment of medical professionals to critical wards such as intensive care units [9]. Staff in CF care centres in the USA experienced salary reductions or layoffs [10]. However, advancements in technology enabled the transformation of face-to-face CF clinics onto online platforms for providing CF care.

The chronic nature and deteriorating health condition of PWCF, along with the inconvenience of CF therapy in general, has often led to higher rates of anxiety and depression among PWCF than in the general population [11]. With CF patients already facing the burden of their health condition, new challenges and uncertainties of the COVID-19 pandemic may have further contributed to the deterioration of their mental health. Thus, it is crucial to address mental health concerns along with physical ones [11].

To the best of the author’s knowledge, this is the first national survey conducted specifically among PWCF that addresses the impact of the COVID-19 pandemic on employment-related aspects. There has been no other study conducted in Ireland addressing a similar range of outcomes.

The aim of this study was to determine the impact of the first 6 months of the COVID-19 pandemic on the lives of PWCF in relation to hospital visits, use of telemedicine, employment, and mental well-being.

## Methods

CF Ireland, the key advocacy organisation for PWCF in Ireland, developed an online self-administered questionnaire with questions adapted from other surveys that were being concurrently conducted for comparative purposes. The surveys from which references were taken for the questions are as follows: Corona Citizens Science project (Waves 1 and 2) conducted by the University of Galway, Dublin City University and the Insight SFI Research Centre for Data Analytics, The Eurordis Rare Barometer Covid Survey, EU survey 2020, The Central Statistics Office, Ireland “Social Impact of COVID-19 Survey April 2020”, and the RAND 36-item Short Form Survey Instrument (SF-36) [12–15]. The questionnaire contained questions on demographics, sources of information about COVID-19, deferral of hospital visits, precautions taken, cocooning, impact on employment, telemedicine, and impact on mental health. Mental health questions have been derived from the SF-36 questionnaire [15]. A pilot study was undertaken. The questionnaire was uploaded on SmartSurvey UK, a General Data Protection Regulation (GDPR) compliant survey tool for a period of 6 weeks. The survey was hosted by CF Ireland and advertised widely through various social media platforms including Twitter, Instagram, Facebook, and PWCF WhatsApp groups. A copy of the participant information leaflet was made available on the CF Ireland website for participants to review. After reading the participant information leaflet, a tick was obtained as consent from participants for both data collection and processing of information.

The questionnaire was made available only to those PWCF resident in Ireland and aged 18 or older. Individuals who reported being under the age of 18 years or living outside of Ireland during the completion of the questionnaire were excluded.

At the time of the survey, 759 adults aged > 18 years were registered with CF Registry of Ireland; thus, 119 PWCF respondents accounted for 16% of the total CF population. Power analysis was not conducted for this study.

The data analysis was conducted by the research team from University College Dublin. Pearson’s chi-squared test was used to compare the impact of COVID-19 on CF care of PWCF depending on age and gender. Unadjusted and multivariable logistic regression were used to determine independent factors associated with the deferral of hospital visits and mental health effects. Analysis was undertaken using SPSS Version 24.

The study was approved by the National Research Ethics Committee for COVID-19 (20-NREC-COV-090).

## Results

During the 6-week period, a total of 119 completed responses were recorded. Sixty-one percent were females and 39% were males; 54% were < 35 years. More than half of the participants (65.5%) were residing in an urban location.

The results for deferral of hospital visits are shown in Table 1. A total of 56 (47.5%) respondents indicated a deferral of hospital visits for CF care. The period of delay ranged from 1 to 6 months. A majority had a deferral of at least 3 months (74%) with 26% deferring for 6 months. Among those who deferred, the key reasons were fear of encountering coronavirus (69.8%) and a smaller proportion found the hospital unit closed (11.5%).

**Table 1:** Deferral of hospital visits by adults with CF

**Variable**	**Adults with CF** *N* **(%)**
**Deferral of hospital visits**	
Yes	56 (47.5)
**Duration of deferral**	**56**
1 month	11 (20.4)
2 months	8 (14.8)
3 months	12 (22.2)
4–6 months	9 (16.7)
6 months	14 (25.9)
**Reasons for deferring**	
**Hospital unit was closed**	**52**
Yes	6 (11.5)
No	46 (88.5)
**Fear of COVID-19**	**53**
Yes	37 (69.8)
No	16 (30.2)

Table 2 shows the results of a logistic regression of factors associated with deferral of hospital visits. Participants aged < 35 years were more likely to defer hospital visits than their older counterparts when adjusted for gender and location (55.6% vs 44.4%; adjusted OR: 2.17; 95% CI: 1.00–4.69). No significant association was observed between gender or location and deferral of hospital visits.

**Table 2:** Logistic regression: factors associated with deferral of hospital visits

**Variable**	**Deferral of hospital visits**					
	**Yes**	**No**	**Univariate OR (95% CI)**	***P* value**	**Multivariate OR (95% CI)**	***P* value**
**Age**	**56**	**62**				
< 35 years	35 (55.6)	28 (44.4)	2.02 (0.97–4.23)	0.061	2.17 (1.00–4.69)	0.05
> 35 years	21 (38.2)	34 (61.8)	1.0		1.0	
**Gender**	**56**	**61**				
Female	34 (47.9)	37 (52.1)	1.00 (0.47–2.10)	0.995	0.95 (0.44–2.05)	0.95
Male	22 (47.8)	24 (52.2)	1.0		1.0	
**Location**	**56**	**62**				
Urban	36 (46.8)	41 (53.2)	1.32 (0.63–2.78)	0.458	1.06 (0.48–2.32)	0.88
Rural	20 (48.8)	21 (51.2)	1.0		1.0	

Table 3 summarises the impact of COVID-19 on the CF care of PWCF based on age and gender. During the pandemic, a non-significantly greater number of older PWCF (aged ≥ 35 years) deferred their rehabilitation therapies (36% vs 29%)), general practitioner (GP) appointments (40% vs 32%) and diagnostic test appointments (50% vs 40%). A non-significantly greater proportion of PWCF aged < 35 years experienced interruption in hospital-based medical services as compared to their older counterparts (60% vs 50%).

**Table 3:** Impact on CF care of PWCF based on their age and gender

** Postponed/cancelled**				
** Based on age of adults with CF**				
**Variable**	**All** ***N* (%)**	** < 35 years** ***N* (%)**	≥ **35 years** ***N* (%)**	***P* value**
Rehabilitation therapies	9 (32.1)	5 (29.4)	4 (36.4)	0.70
Medical care at home	2 (8.0)	1 (6.3)	1 (11.1)	0.67
Medical care at hospital	25 (56.8)	18 (60.0)	7 (50.0)	0.53
Transplant	2 (11.7)	1 (12.5)	1 (11.1)	0.93
Surgery	2 (11.7)	1 (12.5))	1 (11.1))	0.93
Appointment with GP	13 (35.1)	7 (31.8)	6 (40.0)	0.61
Diagnostic tests	16 (45.7)	6 (40.0)	10 (50.0)	0.56
**Based on gender of PWCF**				
**Variable**	**All** ***N* (%)**	**Female** ***N* (%)**	**Male** ***N* (%)**	***P* value**
Rehabilitation therapies	9 (32.1)	5 (33.3)	4 (30.7)	0.89
Medical care at home	2 (8)	2 (13.3)	0 (0.0)	0.23
Medical care at hospital	25 (56.8)	20 (64.5)	5 (38.5)	0.11
Transplant	2 (11.7)	2 (22.2)	0 (0.0)	0.16
Surgery	2 (11.7)	1 (11.1)	1 (12.5)	0.93
Appointment with GP	13 (35.1)	7 (29.1)	6 (46.1)	0.30
Diagnostic tests	16 (45.7)	11 (50.0)	5 (38.5)	0.51

Comparing PWCF based on gender, a non-significantly higher proportion of females deferred their hospital-based medical care (65% vs 39%) and diagnostic test appointments (50% vs 38.5%) due to COVID-19. COVID-19 had a minimal impact on PWCF surgery based on gender.

**Fig. 1:**
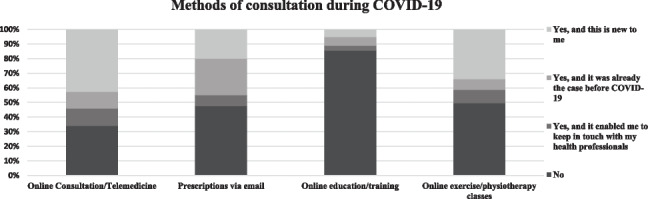
Consultation methods by attitude and previous exposure to methods of consultation during COVID-19

**Fig. 2:**
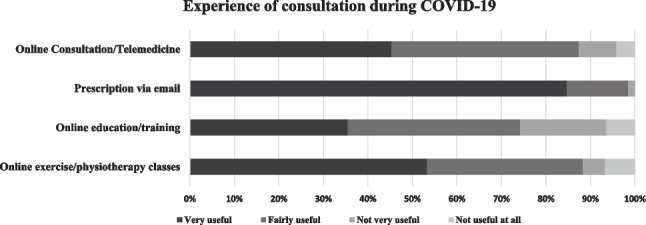
Experience and usefulness of each mode of consultation

Figure 1 shows the various ways through which health professionals were approached during the pandemic, and Fig. 2 shows the usefulness of each mode of consultation. Online consultation was new to approximately half of the participants (47.6%) and the majority found it useful. Overall, 53% received their prescriptions via email, and of those who did, more than 80% of them found it beneficial for them. Small numbers of PWCF used online education or online physiotherapy classes, but those who did found it very helpful.

Among those participants who worked, 41.7% worked from home during the pandemic (Table 4). Very few worked on-site, but this was more common among younger PWCF than older ones (9.6% vs 1.9%). Most employers (91.7%) were sympathetic to the participants who worked from home. More PWCF aged ≥ 35 years stopped working as they were cocooning as compared to their younger counterparts. Over one-third of employers (36.8%) were considered unsympathetic to PWCF who were not working as were cocooning.

**Table 4:** Impact on employment of PWCF based on age and sympathy of employers during the pandemic

	**Based on age**				**Sympathy of employers**	
**Variable**	**Total** ***N* (%)**	** < 35 years** ***N* (%)**	≥ **35 years** ***N* (%)**		**Yes** ***N* (%)**	**No** ***N* (%)**
Worked from home	48 (41.7)	25 (39.7)	23 (44.2)		44 (91.7)	4 (8.3)
Worked onsite as an essential worker	3 (2.6)	3 (4.8)	0 (0.0)		3 (100.0)	0 (0.0)
Worked onsite but not as an essential worker	4 (3.5)	3 (4.8)	1 (1.9)		3 (100.0)	0 (0.0)
Not working as, I am (temporarily) laid off	5 (4.3)	4 (6.3)	1 (1.9)		2 (50.0)	2 (50.0)
Not working due to other reasons	24 (20.9)	11 (17.5)	13 (25.0)		1 (50.0)	1 (50.0)
Not working as, I am cocooning	22 (19.1)	11 (17.5)	11 (21.2)		12 (63.2)	7 (36.8)
Other	9 (7.8)	6 (9.5)	3 (5.8)		1 (50.0)	1 (50.0)

Table 5 shows logistic regression results assessing the impact of COVID-19 on the mental health of PWCF. After adjustment for gender and employment status, PWCF aged < 35 years were more likely to report feeling “nervous” (OR: 3.28; 95% CI: 1.23–8.71), “nothing could cheer them up” (OR: 3.24; 95% CI: 1.07–9.84), and “tired” (OR: 2.76; 95% CI: 1.21–6.27) compared to those ≥ 35 years. On the contrary, a greater proportion of PWCF aged ≥ 35 years felt “calm and peaceful” (OR: 2.25; 95% CI: 1.02–4.93) and “happy” (OR: 2.30; 95% CI: 1.05–5.07) as compared to the younger PWCF. When adjusted for age and gender, PWCF who were employed were 2.24 times more likely to feel energetic as compared to those who were unemployed (OR: 2.24; 95% CI: 1.02–4.91).

**Table 5:** Logistic regression showing psychological health of PWCF when adjusted by age, gender, and work

**Variable**	**All/most/a good bit of the time**	**Some/a little/none of the time**	**Adjusted OR**	**95% CI**	***P*** **value**
**During the period of pandemic restrictions, you felt full of life**					
**Age**	**61**	**57**			
< 35 years	27 (42.2)	37 (57.8)	0.47	0.22–1.02	0.06
≥ 35 years	34 (63.0)	20 (37.0)	1.0		
**Gender**	**61**	**56**			
Female	38 (53.5)	33 (46.5)	1.18	0.54–2.58	0.69
Male	23 (50.0)	23 (50.0)	1.0		
**Work**	**58**	**56**			
No	26 (44.1)	33 (55.9)	0.57	0.26–1.22	0.15
Yes	32 (58.2)	23 (41.8)	1.0		
**During the period of pandemic restrictions, you felt a very nervous person**					
**Age**	**28**	**87**			
< 35 years	20 (32.8)	41 (67.2)	3.28	1.23–8.71	0.02*
≥ 35 years	8 (14.8)	46 (85.2)	1		
**Gender**	**28**	**86**			
Female	19 (27.5)	50 (72.5)	1.50	0.58–3.90	0.41
Male	9 (20.0)	36 (80.0)	1		
**Work**	**27**	**84**			
No, did not work	16 (27.6)	42 (72.4)	1.74	0.69–4.34	0.24
Yes, worked	11 (20.8)	42 (79.2)	1		
**During the period of pandemic restrictions, you experienced that nothing could cheer you up**					
**Age**	**21**	**93**			
< 35 years	16 (26.2)	45 (73.8)	3.24	1.07–9.84	0.04*
≥ 35 years	5 (9.4)	48 (90.6)	1		
**Gender**	**21**	**92**			
Female	13 (19.1)	55 (80.9)	1.06	0.37–3.00	0.91
Male	8 (17.8)	37 (82.2)	1		
**Work**	**20**	**90**			
No, did not work	13 (22.8)	44 (77.2)	2.18	0.77–6.22	0.15
Yes, worked	7 (13.2)	46 (86.8)	1		
**During the period of pandemic restrictions, you have been calm and peaceful**					
**Age**	**47**	**68**			
< 35 years	20 (32.8)	41 (67.2)	1		0.04*
≥ 35 years	27 (50.0)	27 (50.0)	2.25	1.02–4.93	
**Gender**	**47**	**67**			
Female	26 (37.7)	43 (62.3)	0.78	0.35–1.74	0.55
Male	21 (46.7)	24 (53.3)	1		
**Work**	**47**	**64**			
No, did not work	21 (36.2)	37 (63.8)	0.55	0.25–1.21	0.14
Yes, worked	26 (49.1)	27 (50.9)	1		
**During the period of pandemic restrictions, you had a lot of energy**					
**Age**	**52**	**63**			
< 35 years	23 (37.7)	38 (62.3)	0.55	0.25–1.20	0.13
≥ 35 years	29 (53.7)	25 (46.3)	1		
**Gender**	**52**	**62**			
Female	31 (44.9)	38 (55.1)	0.80	0.36–1.77	0.58
Male	21 (46.7)	24 (53.3)	1		
**Work**	**48**	**63**			
No, did not work	20 (34.5)	38 (65.5)	1		
Yes, worked	28 (52.8)	25 (47.2)	2.24	1.02–4.91	0.04*
**During the period of pandemic restrictions, you felt downhearted and blue**					
**Age**	**23**	**92**			
< 35 years	15 (24.6)	46 (75.4)	1.76	0.66–4.66	0.26
≥ 35 years	8 (14.8)	46 (85.2)	1		
**Gender**	**23**	**91**			
Female	13 (18.8)	56 (81.2)	0.78	0.30–2.03	0.61
Male	10 (22.2)	35 (77.8)	1		
**Work**	**22**	**89**			
No, did not work	13 (22.4)	45 (77.6)	1.45	0.55–3.79	0.45
Yes, worked	9 (17.0)	44 (83.0)	1		
**During the period of pandemic restrictions, you have been a happy person**					
**Age**	**63**	**52**			
< 35 years	28 (45.9)	33 (54.1)	1		
≥ 35 years	35 (64.8)	19 (35.2)	2.30	1.05–5.07	0.04*
**Gender**	**63**	**51**			
Female	37 (53.6)	32 (46.4)	0.94	0.42–2.09	0.88
Male	26 (57.8)	19 (42.2)	1		
**Work**	**62**	**49**			
No, did not work	29 (50.0)	29 (50.0)	0.58	0.27–1.28	0.18
Yes, worked	33 (62.3)	20 (37.7)	1		
**During the period of pandemic restrictions, you felt tired**					
**Age**	**64**	**50**			
< 35 years	40 (65.6)	21 (34.4)	2.76	1.21–6.27	0.02*
≥ 35 years	24 (45.3)	29 (54.7)	1		
**Gender**	**63**	**50**			
Female	36 (52.9)	32 (47.1)	0.65	0.28–1.49	0.31
Male	27 (60.0)	18 (40.0)	1		
**Work**					
No, did not work	38 (65.5)	20 (34.5)	2.56	1.14–5.74	0.02*
Yes, worked	23 (44.2)	29 (55.8)	1		

Data are presented as numbers (percentage)

^*^P value less than 0.05

## Discussion

The COVID-19 pandemic has created a global public health emergency, and public health restrictions have been in place in most countries [16]. This study was designed to assess the impact of COVID-19 and associated restrictions on PWCF. Our study showed that a large proportion of PWCF deferred their hospital visits. The major reason for deferral was fear of contracting COVID-19, and in a small number of cases, the hospital CF unit was closed. This deferral rate among those aged < 35 years was almost twice than in those ≥ 35 years. However, no such pattern was highlighted during this study with respect to gender and location of the PWCF. Deferrals also impacted rehabilitation therapies, medical care at the hospital, and appointments with GP. A non-significantly greater proportion of respondents aged ≥ 35 years had to put off their diagnostic tests as compared to those aged < 35 years. Deferral of a hospital visit is of great concern as a routine examination is key to early intervention in infective respiratory exacerbations and other CF-related diseases. Online consultation method has been well-received by PWCF who have found it to be a convenient alternative for aspects of multidisciplinary care [17]. Many PWCF found online consultation new and were satisfied with this method. Prescription through email was also popular among study participants. Most countries introduced similar measures to reduce the transmission of COVID-19. Havermans et al. reported cancellation of hospital appointments by patients in Belgium due to the fear of infection from hospitalised COVID-19 patients [18]. The CF team in Milan cancelled all routine clinic face-to-face appointments to avoid unnecessary hospital visits and virus spread [19]. A Swiss study conducted during the pandemic on 327 subjects found that half of the outpatient clinic appointments of PWCF were either cancelled/postponed due to lockdown [20]. Another Italian study, conducted by Nobili et al., found that patients who were followed up at their clinics tried to avoid unnecessary hospital access and stayed at home as recommended, delaying their scheduled hospital visits [21]. The Eurordis Rare Disease COVID-19 survey report, which included 6945 respondents from 36 European countries and covered 1250 rare disease types, including CF, found that almost half of the respondents did not visit the hospital due to fear of contracting COVID-19; 34% reported that they were not allowed to visit the hospital if their health problem was not related to COVID-19; and 25% reported that their hospital or rare disease care provider unit was closed [13].

Online medical consultations were introduced and 87.8% of PWCF found them useful. Other countries followed suit; a study from Western Australia showed similar findings, with more than 90% of participants with CF strongly agreed/agreed that teleconsultation could be an excellent way of managing CF care [22]. A further study of telehealth performed at Virginia Commonwealth University Adult CF Center showed that 100% of the patients considered access to telehealth for improved care during the pandemic, and 80% supported telehealth implementation in the future in VCU CF Center [23]. A study in Milan documented the cancellation of routine appointments by the CF team who monitored the clinical conditions of patients via phone calls or emails [19]. According to the Eurordis Rare Disease COVID-19 survey report, 50% of the participants engaged in online consultations. Among those who did, 90% found online consultation/telemedicine services, and online education tools designed to assist in managing their rare disease themselves beneficial [13].

The Medicinal Products (Prescription & Control of Supply) (Amendment) Regulations 2020 and the Misuse of Drugs (Amendment) Regulations 2020 were signed into law in March 2020 as part of health-related initiatives enforced in Ireland to curb the viral spread [24]. They introduced the “national electronic prescription transfer system” which allows a prescription to be electronically transferred from the health care professional directly to the pharmacist [25, 26]. This was welcomed by the CF community, with 88% in our study in favour. Repeat prescriptions emailed directly to pharmacy will be continued.

COVID-19 significantly increased the unemployment rate, and decreased hours of work and labour force participation during that period [27]. In our study, it was observed that a greater proportion of older adults stopped working while they were cocooning. Also, over one-third of employers were perceived as unsympathetic towards PWCF who were not working as cocooning. Those who were employed were more energetic and happier as compared to those who were not employed. No significant difference by gender was observed.

The key mental health issues faced by the majority of PWCF during the pandemic were increased levels of nervousness and tiredness. PWCF aged < 35 years experienced more mental distress during the pandemic as compared to the older group, particularly in terms of nervousness and feeling downhearted. Cocooning was recommended at the start of the pandemic by the Irish Government for older people and those considered highly vulnerable to COVID-19, including those living with CF [4]. Studies from several countries including Turkey, the United Kingdom (UK), Belgium, and Italy showed similar findings with high levels of anxiety and depression among PWCF and their families [19, 28, 29]. From a general population perspective, an Irish study of 1000 people in March and April 2020 (during the initial restrictions) found that 41% of respondents felt lonely, 23% recorded depression, 20% reported anxiety, and 18% reported clinically relevant post-traumatic stress [30]. Another survey conducted by the Central Statistics Office in Ireland, titled “Social Impact of COVID-19 Survey April 2020”, reported that 35.5% of respondents from general Irish households reported feeling very nervous, 32.4% felt downhearted and depressed, and 26.6% felt lonely “at least some of the time” in April 2020 [14]. Those with CF were deemed to be in the ‘highly vulnerable’ group, which confers an additional psychological burden.

A limitation of this study is that this was a self-reported study of PWCF recruited through social media and the CF Ireland website. Findings of our study however concur with other authors internationally. This study was conducted at the end of the second wave of COVID-19 in Ireland, a time when there was talk of vaccines, but all were in the trial stages with no definite hope yet. Future work should look at how the rollout of vaccination may change the psychological wellbeing of adults with CF.

## Conclusion

Online consultation worked in part during the peak of the pandemic period and may be of some value going forward. However, virtual CF care, although a convenient method, is suboptimal for complete ongoing CF care [10]. New prescription via email was a COVID pandemic change due to the increase in telemedicine; as in-person clinical care reopened, this is less of a requirement but repeat prescriptions can continue to be available from pharmacies [31]. Mental health was also affected during the pandemic; the role of the psychologist in CF care is already well recognised, but our findings suggest that there will be a greater need for these services in the short-term and possibly long-term future.
